# Overcoming the Challenges to Enhancing Experimental Plant Biology With Computational Modeling

**DOI:** 10.3389/fpls.2021.687652

**Published:** 2021-07-20

**Authors:** Renee Dale, Scott Oswald, Amogh Jalihal, Mary-Francis LaPorte, Daniel M. Fletcher, Allen Hubbard, Shin-Han Shiu, Andrew David Lyle Nelson, Alexander Bucksch

**Affiliations:** ^1^Donald Danforth Plant Science Center, St. Louis, MO, United States; ^2^Warnell School of Forestry and Natural Resources and Institute of Bioinformatics, University of Georgia, Athens, GA, United States; ^3^Department of Systems Biology, Harvard Medical School, Boston, MA, United States; ^4^Department of Plant Sciences, University of California, Davis, Davis, CA, United States; ^5^Bioengineering Sciences Research Group, Department of Mechanical Engineering, School of Engineering, Faculty of Engineering and Physical Sciences, University of Southampton, Southampton, United Kingdom; ^6^Department of Plant Biology and Department of Computational Mathematics, Science, and Engineering, Michigan State University, East Lansing, MI, United States; ^7^Boyce-Thompson Institute, Cornell University, Ithaca, NY, United States; ^8^Department of Plant Biology, University of Georgia, Athens, GA, United States; ^9^Institute of Bioinformatics, University of Georgia, Athens, GA, United States

**Keywords:** computational modeling, mathematical modeling, bioinformatics, collaboration, experimental design

## Abstract

The study of complex biological systems necessitates computational modeling approaches that are currently underutilized in plant biology. Many plant biologists have trouble identifying or adopting modeling methods to their research, particularly mechanistic mathematical modeling. Here we address challenges that limit the use of computational modeling methods, particularly mechanistic mathematical modeling. We divide computational modeling techniques into either pattern models (e.g., bioinformatics, machine learning, or morphology) or mechanistic mathematical models (e.g., biochemical reactions, biophysics, or population models), which both contribute to plant biology research at different scales to answer different research questions. We present arguments and recommendations for the increased adoption of modeling by plant biologists interested in incorporating more modeling into their research programs. As some researchers find math and quantitative methods to be an obstacle to modeling, we provide suggestions for easy-to-use tools for non-specialists and for collaboration with specialists. This may especially be the case for mechanistic mathematical modeling, and we spend some extra time discussing this. Through a more thorough appreciation and awareness of the power of different kinds of modeling in plant biology, we hope to facilitate interdisciplinary, transformative research.

## Introduction

Generating knowledge requires the integration and contextualization of information: “A collection of facts is no more a science than a heap of stones is a house” (Henri Poincaré). The increasing availability of data provides opportunities as well as challenges to integrate information and properly describe complex biological systems. ***Mathematical modeling*** is the process of describing complex systems in a logically consistent and explicit manner using a quantitative framework (Nijhout et al., 2015). Such models can generate testable hypotheses by relating possible mechanisms and relationships to observable, measurable phenomena (Bennett et al., 2019). In addition, models are used to identify non-intuitive relationships, emergent properties, and the conditions under which phenomena arise. In the first half of the paper, we address questions that plant biologists may have about modeling (sections 1–4), followed by challenges to overcome hurdles (sections 5–9). Here, we begin by dividing the field of mathematical modeling in plant biology into two categories: pattern-finding and mechanistic mathematical models (section 1). We then address how these types of models are used in different subfields of plant biology, and how pattern and mechanistic mathematical models complement each other (section 2) (Bucksch et al., 2017; Passot et al., 2019). Then we further describe the scientific value of modeling (section 3). We then specifically focus on modeling approaches that are under-used in plant biology (section 4). In the second half, we identify the current challenges and potential solutions to broadening engagement with models in plant biology, such as the required expertise and the difficulty finding modeling collaborators.

## Review of Modeling in Plant Biology

### 1. Types of Models

**Figure 1:**
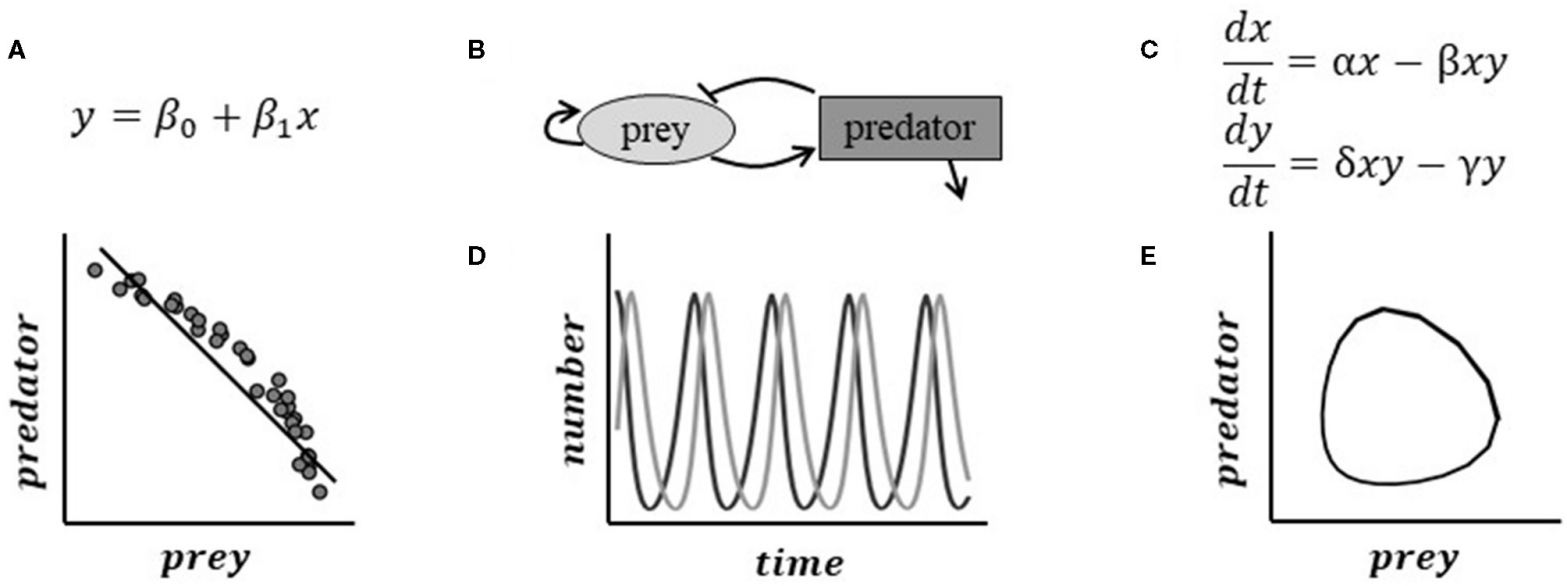
Pattern and mechanistic models approach the same problem in different ways, producing different inferences. Here, we use the system of a predator and its prey for illustration. **(A)** A pattern model's analysis of data might show that generally the number of prey increase as the number of predators decrease. This result might be non-intuitive and difficult to interpret on its own. **(B)** Hypothesized relationships between the predator and prey suggest mechanisms that may be driving the dynamics. **(C)** Mechanistic mathematical models represent the interactions driving this process using a system of equations. Simulation of the theoretical system can help us understand non-intuitive results. **(D)** The Lotka-Volterra predator-prey model predicts a cyclical feedback pattern between predator and prey. Sampling randomly from the true relationship **(E)** produces the data snapshot in **(A)**.

To facilitate communication, we divide computational models roughly by their utility to plant biology—to study patterns or mechanisms. ***Pattern models*** test hypotheses about spatial, temporal, or relational patterns between system components (e.g., individual plants, proteins, genes). The mathematical representation of these hypotheses is based on assumptions about the data and statistical properties (such as regulatory network topology Tyson et al., 2019 or appropriate probability distributions for phenotypic data Kirkpatrick et al., 2016). Thus, pattern models are typically more “data-driven,” i.e., involving finding patterns from the data. Pattern models draw from many disciplines such as bioinformatics, statistics, and machine learning (Zakharova et al., 2019). Many areas of plant biology are studied with pattern models, including the development of genome annotations, phenomics, proteomics, and metabolomics. Big data problems are often addressed using methods such as dimension reduction (e.g., clustering of expression data), latent feature extraction, or machine learning (e.g., neural networks) (Hériché et al., 2019). Spatially-derived patterns, such as plant anatomical structures, are typically addressed using topology and geometry (Amézquita et al., 2020). The identified patterns (e.g., correlation between x and y in Figure 1) constrain the set of possible hypotheses about mechanistic relationships that can explain these observed patterns.

***Mechanistic***^1^ ***mathematical models*** describe the underlying chemical, biophysical, and mathematical properties within a biological system to predict and understand its behavior mechanistically (Keurentjes et al., 2011). Examples of some well-known mechanistic relationships include density-dependent degradation that produces exponential decay; the law of mass-action in biochemical kinetics; and logistic population growth. Mechanistic mathematical models are descriptions of real systems but must balance realism with parsimony. Parsimony refers to the simplest but necessary core processes and components (e.g., Occam's razor)—itself a knowledge-generating process. Parsimonious models permit the study of relationships between the system's hypothesized structure and the resulting behavior of the system (gomez and Ginovart, 2009). Fully realistic models are rarely possible, given the number of biological unknowns, and present computational challenges. For pattern models, parsimony is not always an issue. Some statistical approaches may penalize high-dimensional models, but other approaches (such as neural nets) may use thousands of parameters.

Many mechanistic mathematical models are ordinary differential equations (ODEs, Figure 1). In essence, these models specify how components change with respect to time or space, such as biochemical reactions changing the concentration of proteins. The reactions between components are controlled by one or more rate parameters. These parameters represent the strength and directionality of an interaction or reaction, and may be estimated from data or literature. In addition to specific measurements, we can compare model predictions to our conceptual understanding of how the system works. Different mathematical formulations can be used to describe different biological properties, and affect how the inputs influence the model components.

Mechanistic mathematical models permit the rigorous study of our hypotheses about phenomena without data. For example, in Figure 1, a mechanistic mathematical model could predict what gathered data might look like by simulating the impact of predator or prey interactions over a suite of possible values and population sizes. The example in Figure 1 can also predict the expected data given the experimental sampling times and variability of the system. Through this mechanistic mathematical allows for the elimination of possibilities based on current understanding of the system before data are collected—even guiding the experimental design (Braniff and Ingalls, 2018). Mechanistic mathematical models have yet to reach their full potential in plant biology (Holzheu and Kummer, 2020). This is at least partly due to the challenges associated with the lack of quantitative education in biology curriculum (Bialek, 2004) and communicating mathematical representations of the models to biologists (Fawcett and Higginson, 2012).

### 2. Modeling Approaches in the Plant Sciences

While pattern and mechanistic mathematical models complement each other, there are far fewer mechanistic mathematical models being used in plant biology (with a few exceptions). Several limitations to their adoption exist - but before we address these issues, we will establish why mechanistic mathematical modeling is relevant to you and your research.

#### 2.1. Gene Expression

Pattern models are widely used in plant science to study genetics and gene expression. These models exploit statistical detection of patterns, often through analysis of variability, combined with computational algorithms that allow their application to large datasets across genotype and time. Currently, one of the most abundant types of data is from RNA sequencing (RNA-seq) approaches. RNA-seq is used to measure transcript abundance at a genome-wide scale, examine degrading RNAs, RNA structure, post-transcriptional modifications, and small RNA populations. Software such as DESeq2 deploy general linearized modeling approaches, often utilizing a negative binomial distribution, to identify genes whose expression changes under the influence of a treatment condition (DESeq2) (Love et al., 2014). Pattern modeling can integrate molecular (e.g., transcript abundance) and physiological phenotypes to predict causal genes underlying a trait of interest through the identification of correlations. For example, transcriptome-wide association studies (TWAS) showed that altered transcript abundance explains half of the variation in a number of metabolic and agricultural traits in maize (Kremling et al., 2019). In addition, pattern models have been used to identify genes that influence phenotypes such as yield through their impact on the metabolome using metabolomics QTL (mQTL) (Wei et al., 2018). Identifying functionally correlated transcripts from small populations of samples, or time series data, are typically performed using pattern modeling approaches such as weighted gene co-expression analyses (WGCNA; Langfelder and Horvath, 2008), or circadian aware statistical models such as JTK_Cycle (Hughes et al., 2010). In the realm of single-cell informatics, statistical models such as Seurat or Monocle allow the tracking of cells along development without a priori knowledge of the specific transcripts that define those processes.

In the analysis of gene expression, pattern models typically look for linear relationships between variation in gene expression across a putative driver of that variation, such as different genotypes. However, the underlying processes that drive plant adaptation and behavior are very nonlinear, and statistical approaches that focus on correlations are limited in their discovery ability (Nijhout et al., 2015). Besides, correlation in pattern models is not causation. Mechanistic mathematical models then come into focus as useful to understand the processes that may be driving what we observe. For example, in a mechanistic mathematical model, developmental timing stochasticity explains “noise” and patterns of gene expression in Arabidopsis roots (Greenwood et al., 2019). This work is a nice example of how patterns and mechanisms inform each other, and we anticipate many more discoveries of this type thanks to the interplay between these models in the future.

#### 2.2. Gene Regulatory Networks

Predicting gene regulatory networks (GRNs) is a core interest in plant systems biology (Haque et al., 2019). Biological responses to internal and external signals are mediated by transcription factors (TFs), some of which regulate the expression of hundreds of genes (Bilu and Barkai, 2005). The structure and dynamics of TF-gene and TF-TF interactions control diverse biological processes ranging from spatial patterning in tissues (Adrian et al., 2015), to stress responses (Song et al., 2016). Due to the sheer number of interacting components, TF-gene interactions are often represented as directed networks (GRNs). The past decades have seen numerous pattern modeling approaches for inferring GRNs (GENIE3, etc, best performers of DREAM4) from a variety of sequencing data (gene expression data from RNAseq, TF occupancy data such ATACseq, etc). Although inferred GRNs have significantly improved our understanding of how plant gene expression is regulated, these GRNs are static and thus limited in providing mechanistic insight into the biological process itself. Static networks cannot be used to explore the temporal dynamics of processes, and fail to capture the interactions between GRN components.

GRNs have been successfully implemented beyond static representations through the incorporation of mechanisms. Mechanistic mathematical models can be generated from data-focused pattern modeling techniques, and these models in turn predict patterns that can be validated (Pratapa et al., 2020). The mechanistic mathematical model represents the GRN as a dynamic network which can be simulated by altering the state (Boolean ON or OFF, i.e., bound or not bound) of each TF in the network—an approach that can accurately capture TF regulatory mechanisms (Albert et al., 2017; Pratapa et al., 2020). In this manner, mechanistic networks can provide insight into various network behaviors and cellular decision-making. Mechanistic mathematical models can also be expanded to include metabolomic components of regulatory networks, such as the Boolean network model of the ABA drought stress regulatory network (Albert et al., 2017). This approach requires extensive curation of genetic and metabolomic activity, but produces a system that predicts a wide variety of mutants on the network behavior.

#### 2.3. Signal Transduction Pathways

Mechanistic mathematical models are popular in this area of plant biology. At this scale, plant biologists are more able to collect temporal data with sufficient time resolution to capture the dynamics of system components. At larger scales, from *in vivo* tissue to organs or whole plants, this may require many sampling points and data types that push the boundaries of existing technologies. Pattern models are rarely applied at this scale of plant biology, often asking questions about spatio-temporal gene expression or regulation patterns (Geng et al., 2013), or developmental patterning (Di Mambro et al., 2017). Models of cellular processes include circadian clock and signaling (Grima et al., 2018), the cell cycle (Roodbarkelari et al., 2010), gene expression (Greenwood et al., 2019), development and cell fate (van Berkel et al., 2013), membrane batteries (Dreyer et al., 2019), photosynthesis (Brian and Hahn, 1987), and carbon flux through metabolic pathways (Allen et al., 2009; Orth et al., 2010).

#### 2.4. Physiology

Dynamic processes are the key phenomena of interest in plant physiology. From how water moves throughout a plant to how plants grow, plant physiology is concerned with the flow and change of matter and energy throughout the plant body. Mathematical modeling is necessary to describe these processes precisely and in detail, thus modeling is popular in this area of plant biology.

The regulation of stomatal aperture is an excellent example of mechanistic modeling in plant physiology. Stomatal aperture controls the rate of carbon dioxide assimilation (and therefore photosynthesis) but also controls the rate of transpiration (and therefore plant water balance). Since these processes ultimately determine the productivity and water use in crops and forests alike, mechanistic quantitative descriptions provided by mathematical models are necessary for agriculture and climate forecasting. The regulation of stomatal aperture is also a microcosm of approaches to plant physiological modeling (Buckley, 2017), ranging from the phenomenological (e.g., Jarvis, 1976; Ball et al., 1987) to the biochemical/reductionist (Hills et al., 2012) to teleonomic/non-reductionist (Cowan and Farquhar, 1977; Manzoni et al., 2013; Wolf et al., 2016; Sperry et al., 2017; Mrad et al., 2019).

A variety of mathematical models for many plant physiological processes have been proposed such as leaf and canopy photosynthesis (Hikosaka, 2016), xylem hydraulics (Mrad et al., 2018), phloem translocation (Stanfield et al., 2019), growth morphology (Prusinkiewicz and Runions, 2012; Sievänen et al., 2014; Prusinkiewicz and Barbier de Reuille, 2018), carbon allocation dynamics (Le Roux et al., 2001; Franklin et al., 2012; De Kauwe et al., 2014; Merganicova et al., 2019), genotype-to-phenotype mapping (Diane et al., 2019), and carbon-to-nitrogen allocation (Chen et al., 1993; Dybzinski et al., 2011; Barillot et al., 2016). In addition to physiological processes, these examples range in biological scale, computational complexity, and mathematical sophistication; while commonalities exist along each of these axes, general statements are difficult. However, this diversity suggests a research program aimed at synthesis and provides an excellent source of ideas for that goal.

#### 2.5. Shape and Morphology

A variety of mathematical techniques from topology and geometry are used to describe plant shape and exploit the analytically common or distinguishing characteristics of shapes as phenotypic traits (Bucksch et al., 2017; Mao et al., 2018). Technically, morphological modeling is realized through image processing as a means to extract plant geometry, segmentation or computer simulation to characterize relations between elements like connectivity and hierarchy of branches, arrangements of cells in a space or location of molecules. The field of morphological modeling seeks to understand how underlying mechanisms, including gene regulatory networks, cellular signaling, organ signaling, and biophysical limitations, interact with physical growth processes, and how this ultimately produces the overall size and shape of different plant organs (Chickarmane et al., 2010; Bucksch et al., 2017; Hong et al., 2018).

Persistent homology is a topological pattern modeling technique that describes a relation between plant morphology and a known expanding mathematical function. For example, a circle that continuously increases its diameter from the center of mass of a leaf outline can record the diameters at which serration of the leaf begins (birth) and ends (death) by tracking the intersections between leaf outline and the circle. In that way, subtle differences in the regularity of serration can be detected and potentially linked to genes controlling the serration pattern (Mao et al., 2018). Similarly, the same technique can be used to quantify the branching complexity of root systems in 2D images by recording loops in the skeleton of the 2D projection. The difference between birth and death diameters allows for insight into size variation within loops and therefore summarizes branching frequencies and root density distribution within the root system in one mathematical construct.

Mechanistic mathematical models can enhance the information content and prediction of shape development. For example, the FiberWalk (Bucksch et al., 2017) characterizes the interaction between elongation and lateral expansion processes of tip-driven growth of a branch. The model predicts that tip-driven growth does not result in an equally thick branch and can not reach all spatial locations, providing mechanistic interpretation of some of the observed variation in measured phenotyping data. Both models and segmented images of plant geometry can be used in mechanistic models of plant functions. This approach is often utilized in root-soil models where the geometry of the root systems are hypothesized to play an important role in the root function (e.g., water and nutrient acquisition and stability) (Dunbabin et al., 2013). For example, in the FiberWalk model, branching was found to be a necessary process to optimize nutrient and water uptake below ground (Bucksch et al., 2014).

#### 2.6. Root-Soil Models

Understanding the structure and growth of roots is important for improving plant productivity. However, the difficulty involved in imaging roots in opaque soil motivates mechanistic mathematical modeling of root growth and the resulting root architecture (Schnepf et al., 2018). These models (often called “root-soil” models) need both a mechanistic description of plant and soil processes to understand the function of root systems. Root-soil models are a good example of mechanistic mathematical models applied across scales (e.g., root branching and the biophysical processes involved in water uptake), as well as the seamless transition from the pattern modeling approach of morphology to mechanistic mathematical modeling.

Factors such as water flow in the xylem, transpiration, and diurnal rhythm often play an important role in root-soil models (Schnepf et al., 2012; Hayat et al., 2020). For example, if water is stored throughout the depth of the soil, deeper rooting growth patterns are preferable; while if the soil has a low water-retention capacity, dense and shallow rooting is preferable (Leitner et al., 2014; Tron et al., 2015). Roots can be represented by the root length/surface density (unit length/surface of root per unit volume of soil) as a function of soil depth and time (Ruiz et al., 2020a; Fletcher et al., 2021) or image-resolved geometries (Ruiz et al., 2020b). The function of root, root hairs and soil aggregate geometries can be studied using image-based modeling (a mechanistic approach) using high-resolution 3D imaging of roots in soil, typically X-ray computed tomography. For example, an image-based model found that root hairs and the root contributed equally to phosphorus uptake due to the larger surface area of the root compared to the root hairs. Image-based modeling can complement root imaging studies by solving the mechanistic mathematical model on the image-derived computational mesh, and comparing model predictions to morphological measurements of the root structure (McKay Fletcher et al., 2020). Root systems which had root tips in close proximity obtained the most additional phosphorus uptake due to organic-acid exudation. In summary, mechanistic mathematical models are also powerful vehicles to incorporate multi-scale processes, heterogeneous data such as soil, and complex geometries and are a future direction of focus for the field (Roeder et al., 2011; Bucksch et al., 2017; Hong et al., 2018; Ruiz et al., 2020b). Additionally, mechanistic models can be coupled with imaging studies and growth models to link observed plant structure to underlying function.

#### 2.7. Whole Plant and Agronomic Traits

Crop models (CM) attempt to describe the development, physiology, yield, and agronomic qualities of crop plants, based on genetics, environment, and management. CMs are used by geneticists and breeders to understand the impacts of genotype and environment on traits such as yields, pathogen resistance, and agronomic quality, or to further the understanding and experimental direction for a crop plant of interest (Asseng et al., 2014). CMs often incorporate a variety of inputs, including nutrient availability, radiation, weather, genetic influences on growth, influences from pests and pathogens, and/or field management practices (Jones et al., 2003; Asseng et al., 2014; Donatelli et al., 2017). To synthesize these complex inputs into a cogent model, crop modelers utilize both mechanistic and pattern models.

CM are unique in that they are neither purely patternistic nor mechanistic, often integrating both. Typically, mechanistic mathematical models are incorporated as “sub-models” of a compartment (such as weather patterns or photosynthesis) within a larger empirical modeling structure, often including pattern modeling components For example, DSSAT (Decision Support System for Agrotechnology Transfer) models simulate crop growth by utilizing mathematical representations of soil and weather relations alongside empirical findings for specific crops' growth habits (Jones et al., 2003). Thinking about CM may be useful to experimentalists learning about mechanistic mathematical models as an “exception that clarifies the rule.” In the future, we expect the field of CM to become increasingly mechanistic, particularly as computational limitations decrease. The *crops in silico* project has begun to visualize and simulate biological processes, from the molecular to ecosystem-scale (Marshall-Colon et al., 2017)^2^. These efforts have the potential of producing fully mechanistic mathematical models, which could inform further experimentation and research directions. Since the two types of models we lay out here are a spectrum, rather than a dichotomy, considering the way that CMs integrate both areas of modeling may be helpful to clarify the conceptual differences.

## Why Modeling Is Useful

### 3. How Models Can Contribute to Your Research

Hopefully you now see some intersection and value in pattern and mechanistic mathematical models with your research. Even so, models are extra, potentially new work involving learning coding, mathematics, and other quantitative theories—what do they bring to the table in general? We argue that mechanistic mathematical models are not only the natural next step to pattern model discoveries widely used already, but also function in a unique manner to advance plant science for four reasons.

1. **Abstraction of complex systems to produce tractable problems** We begin with a list of facts and information. This gets reduced and simplified depending on the research question. This abstraction process can be helpful to enhance our understanding of biology, addressing questions such as the minimum required components to produce a given phenomenon including feedback, oscillatory behavior, or spatial patterning. For example, a mechanistic mathematical model of auxin signaling in the formation of root nodules predicted ‘signature' patterns that allow experimental discrimination between the possible underlying mechanisms driving the behavior (Deinum et al., 2012).
2. **Predicting emergent phenomena** The interesting parts of a system are when you begin to observe unexpected behavior. Such behavior helps us identify the significance of the roles of specific components within a system. Theoretical tools can be applied to mechanistic mathematical models to allow us to make claims about qualitative and emergent behaviors of a system. For example, bifurcation analysis predicts previously unknown protective relationships between pathogens in a model of disease transmission (Chen et al., 2018) or how precipitation regimes give rise to distinct landscape vegetation patterns (Tarnita et al., 2017). Mathematical phenomena like switches, bi-stability, and attractors (Saadatpour et al., 2016; Rata et al., 2018) may produce additional emergent behaviors that otherwise may go unnoticed with standard experimental exploration of the stimulus space. Additional analytical or numerical study can predict “breaking point” or “unrealistic” behavior. If a predicted “breaking point” is not observed experimentally, the model's representation needs to be re-evaluated. This allows us to avoid wondering if “maybe it just wasn't enough of a [stimulus]” when designing experiments.
3. **Suggesting mechanisms not present in our intuition of a system** After formulation of a model describing a system, we may notice that it critically disagrees with our observations. In this case, we may question the suitability of the pattern model for the data without considering the disagreement biologically informative. On the other hand, disagreement between a mechanistic mathematical model and the data often suggests our understanding of the system may be wrong. Mechanistic mathematical models are quantitative representations of our hypotheses. Disagreement between mechanistic mathematical models and data may also predict the existence of relationships not previously considered as critical to producing the phenomena or dynamics of interest, or the mathematical representation is not appropriate. The back-and-forth between quantifying our understanding via mechanistic mathematical models and assessing agreement with data has the potential to produce new biology, new mathematics, and new mathematical biology questions to be pursued.
4. **Integrating knowledge and understanding across system scales** Experimentation reveals how a particular component interacts with other components in the system. Pattern models can reveal these interactions, while mechanistic mathematical models can test them. One of the strongest benefits of mechanistic mathematical modeling is the ability to incorporate multi-disciplinary concepts, such as chemistry (Hills et al., 2012; Dale and Kato, 2016), biophysics (Deinum et al., 2012; Amiri et al., 2019; Dreyer et al., 2019), and multi-scale processes (Feller et al., 2015). Biological systems are necessarily controlled by chemical and physical processes, and in certain cases these effects should not be ignored.

### 4. Mechanistic Mathematical Modeling Is Under-Utilized in Plant Biology

The biggest challenge to the wider adoption of mechanistic mathematical modeling in plant biology is implementation. Indeed, it is often challenging for non-modelers to specify a modeling approach, let alone develop the necessary models. Experience is needed to propose a minimal model of the system, identify the appropriate experimental design, choose an appropriate mathematical representation, and carry out computational and mathematical analysis to study the resulting model.

**Mechanistic mathematical models are usually specific** Pattern models can often be useful as “black-boxes” (e.g., an input of data into a pattern model, an output of a *p*-value). However, mechanistic mathematical models are typically very specific. While mechanistic mathematical models excel at making predictions for a variety of contexts, analysis of a given data set often requires modifications. Their utility results from synthesizing biological concepts into a coherent whole and applying them to specific phenomena or experiments. Mechanistic model development requires an understanding of both the biological system and the mathematics; pattern models can be developed for many applications since correlations in data exist independent of what the data represent. For example, an RNA-seq approach could be applied to any species, for any environmental condition to understand gene expression patterns. A mechanistic mathematical model would need to be specific to the TFs and genes of interest; further, environmental stressors cause changes in different response pathways, necessitating completely different models.

**Model development requires math (to some extent)** Mechanistic mathematical models require math, which may be intimidating—whether you are writing one yourself, or trying to collaborate with a modeler. A good background to mechanistic mathematical modeling includes understanding the theoretical basis as well as its practical relevance to plant biology and their implementation and validation. For example, ODE modeling uses mathematical biology theory such as mass-action kinetics or standard mechanistic equations mentioned earlier in section 1; mathematical concepts from calculus and differential equations; methods to simulate and solve equations; and computational methods to estimate parameters, from least-squares to Bayesian and machine-learning approaches. If you are a biologist interested in mathematics, a good starting point is Ledder et al. (2013).

**Collaborating with mathematicians** Traditionally, mathematicians who developed mechanistic mathematical models were experts in a field of theoretical math. This means that biologists seeking to develop mechanistic mathematical models for their research needed a deep understanding of modeling for a productive collaborative discussion to take place. Alternatively, biological problems would have to reach the ears of applied mathematicians, who then sought out biologists. Fortunately, we now have specialists in computational plant biology, as well as mathematical modelers working on similar phenomena in other biological systems. This greatly reduces, although does not eliminate, collaborative issues.

## Solutions to Common Challenges

### 5. Modeling When You Don't Like Math

**Figure 2:**
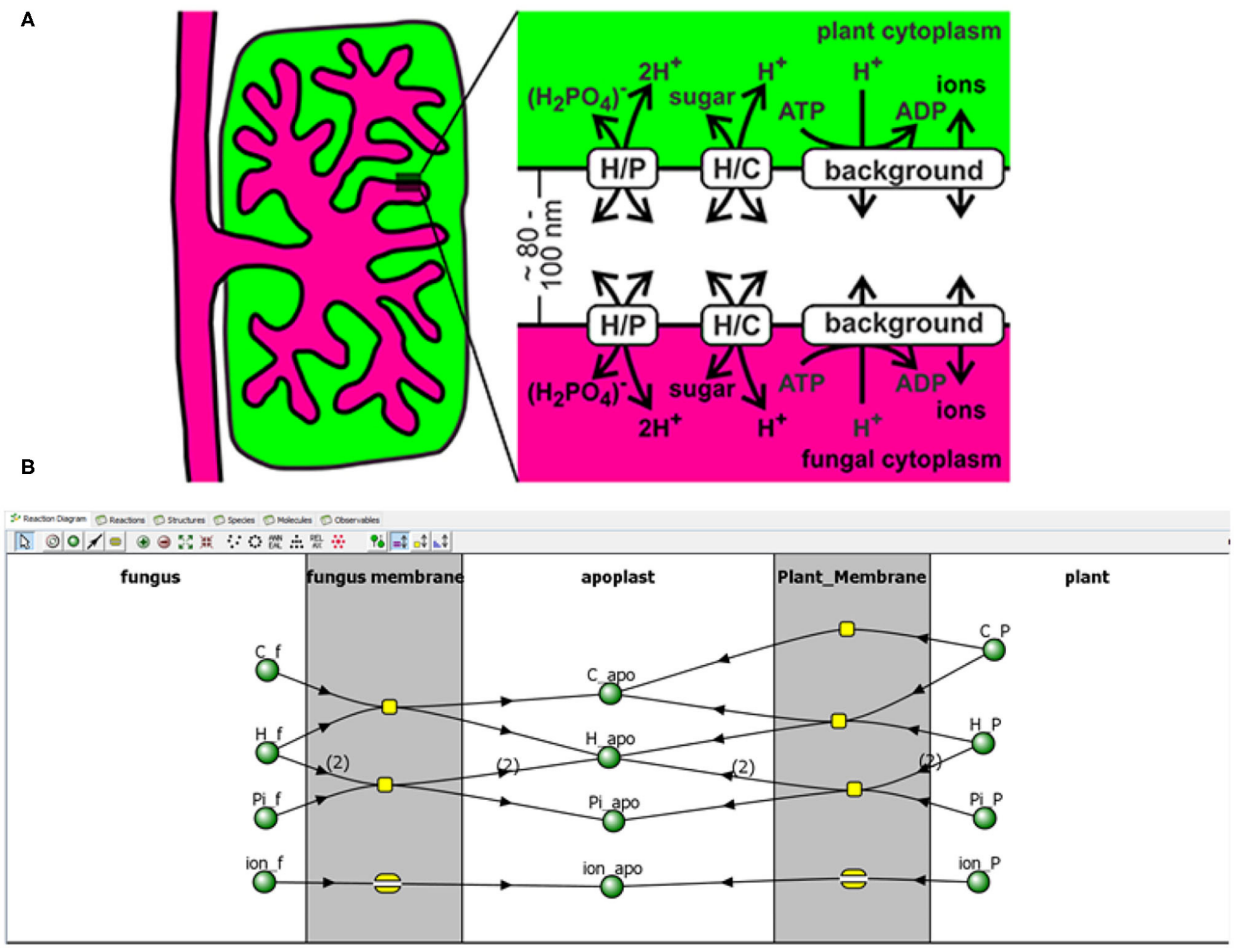
Example of using VCell. A conceptual model of plant mycorrhizal trade is developed **(A)**. A model of this system can be developed in VCell^4^ through a graphical interface **(B)** and text-based descriptions of rules, such as reactions and movement. Reproduced from (Schott et al., 2016). Over 800 published models, including from Schott et al. (2016), are available to run immediately upon installation of VCell.

It's an old stereotype that people go into the field of biology because they don't like math (Wachsmuth et al., 2017). Rest assured—you can still model without doing math. In some cases, models can be developed using software for a wide array of biological systems without an in-depth knowledge in the underlying mathematics, including biochemical questions (COPASI^3^); signaling, cellular, and multicellular questions (VCell^4^ and SBML Hucka et al., 2003); (see Figure 2) and spatial and ecological questions (LANDIS II^5^). These tools automatically translate diagrams and rules into equations, with anywhere from minimal to high levels of coding required. Tools are also developed to study special systems in plant biology, such as stomatal regulation (Hills et al., 2012). While research questions often still require the attention of a modeler, these approaches would certainly help facilitate conversation with, if not totally suffice as the model.

### 6. Finding and Collaborating With Modelers

To facilitate collaboration for those cases where more complex analysis is required, we recognize the importance of the personal connection. Collaborative incubators and workshops have increasingly sprung up to meet this need, such as Finding Your Inner Modeler (FYIM), Probability Meets Biology (Probability meets biology), Quantitative Cell Biology network (QCB Workshops QCBNet), and NIMBioS workshops^6^. However, more work is needed. Math can be scary, and we need human connection. To partially address this we are developing a collaborative website, https://www.initmathbio.com. This website works in conjunction with an open database of participants at previously held collaborative workshops we have held. We hope that with this website, you will be able to describe your problem, obtain feedback from subject matter experts, and find collaborators to jump-start your modeling. Some aspects of collaboration are particularly challenging, and we offer the following suggestions:

1. **Don't assume anything is not important** The experimental assumptions and methods are often just as (if not more) important than the system itself. Models often have to reflect the experimental design as well as the biology of interest (Dale et al., 2016).
2. **Specify research questions and their impact** Why do you want to model your system? What is currently unknown, and what significance does that have to the field? Often experimental biologists want a model for vague reasons (e.g., “surely I have enough data to model”). Hopefully, after reading this paper, you are now aware that modeling comprises a vast array of approaches; a collaboration will be more efficient if a model can be contextualized.
3. **Be patient** The interaction between modelers and experimentalists is a learning process that involves both parties. Modelers must develop domain knowledge relevant to the biological question, while experimentalists need to get familiar with the abstract thinking (simplification) in the modeling approach. Many conversations will be required before model development begins.

### 7. Appreciating How Pattern and Mechanistic Mathematical MODELS Fit Into the Scientific Method

**Figure 3:**
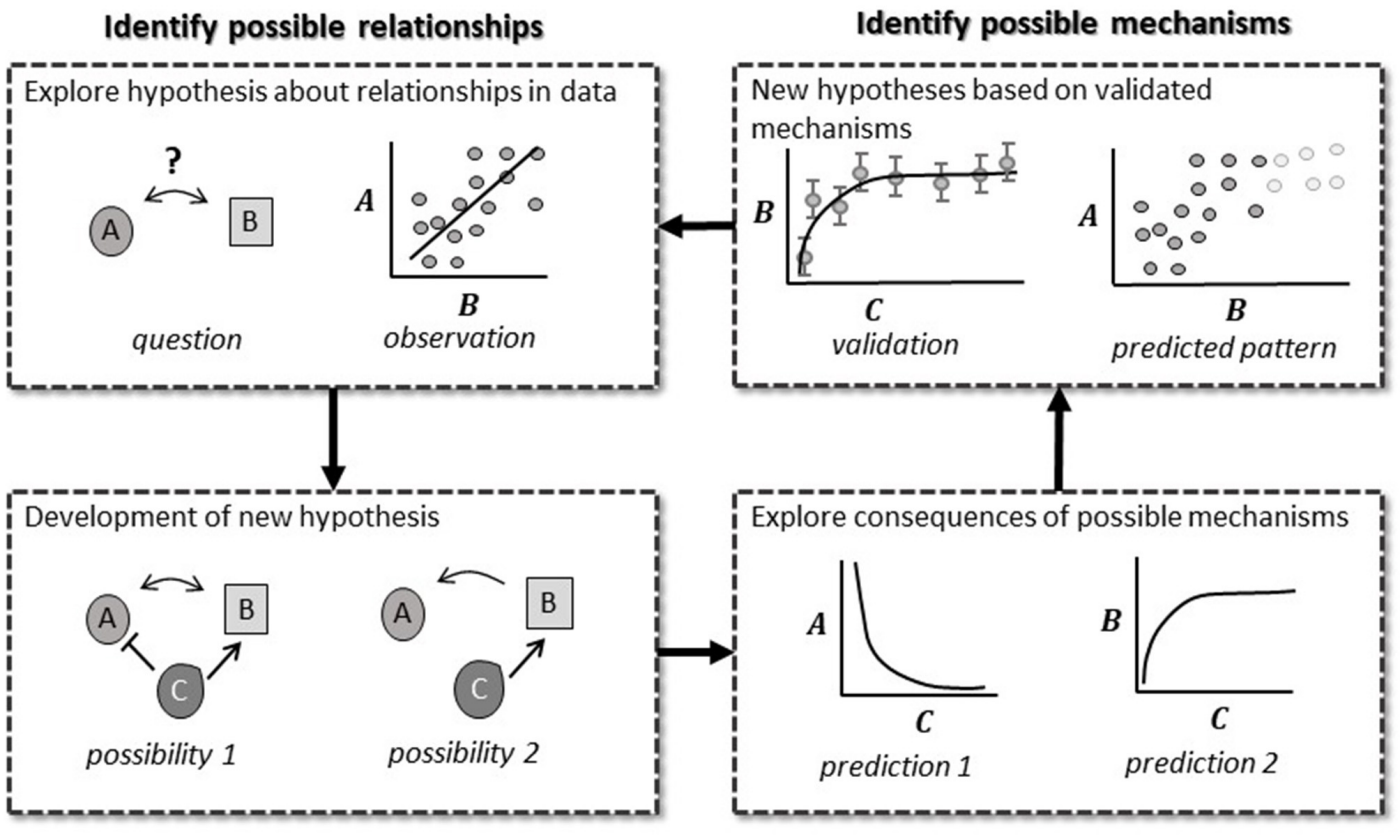
Pattern (L) and mechanistic mathematical models (R) have different strengths that fit into the scientific method. Pattern models test predicted patterns based on biological theory. Observed patterns then allow us to form a more detailed theory on possible mechanisms driving these patterns. Mechanistic mathematical models allow us to identify ways to discriminate between these possibilities. Once validated, the biological theory is updated further, and new patterns are predicted.

Modeling should be a back and forth between model and experiment, and an iterative improvement over previous models in order to answer a question (Figure 3) (Mogilner et al., 2006; gomez and Ginovart, 2009; Tyson and Novak, 2010). This integrative process is called the modeling cycle, and mirrors the scientific method (hypothesis, experiment, evaluation, repeat). The modeling cycle starts with composing a preliminary model of the phenomena of interest (“hypothesis”). The model may be a network of components with interactions based on scientific theory, existing data, or an existing model. The model is compared to experimental data, or used to predict experimental designs where certain outcomes will occur (“experiment”). The resulting model can then be used to adjust our experimental designs to fill knowledge gaps (“evaluation”). The back and forth process between model predictions, *in silico* simulations, and experimentation produces gradually improved models and depth of biological inference that lets the utility of modeling shine (Mogilner et al., 2006; Tyson, 2007; Keurentjes et al., 2011; Ratushny et al., 2011; Brodland, 2015; Long, 2019; Holzheu and Kummer, 2020).

Rather than thinking of mathematical models as black boxes that data is shoved into, plant biologists of the future need to “move seamlessly between computational and cell biology” to understand how models predict results, drive design, and produce hypotheses (Short, 2009). This is challenging due to social and technical difficulties associated with quantitative proficiency. Fortunately, it has been shown that math appreciation increases with its utilization (Marsteller, 2010; Chen et al., 2018). Recent emphasis on integrative and translational research and large collaborative groups or hiring clusters facilitate the collaborative, “non-specialized” nature of modern science and facilitate those transitions.

### 8. Consulting Modelers Before Experiments Take Place

One of the most under utilized benefits of models is their ability to predict interesting behavior based on a preliminary model. Although this collaborative approach necessitates additional upfront work, or the willingness of the experimental biologist to get their hands dirty with math, the outcome is far preferable to an experiment that won't let us fully interrogate the patterns or mechanisms in question.

One approach to implementing this successfully is ***model-based experimental design***. Designing an experiment that will facilitate modeling and maximize its inferential power isn't always intuitive, and we recommend consulting a modeler during the design process to ensure the model provides insight to the research question (Drubin and Oster, 2010; Braniff and Ingalls, 2018). Other considerations of experimental design include how the existing model can be improved, via its structure, parameter estimates, or assumptions. There are different methods of evaluating its quality—such as frequentist or Bayesian statistical approaches (Barnes et al., 2011), control theory (Thomas et al., 2019), optimization theory (Wang et al., 2010), sensitivity analyses (Barnes et al., 2011; Heinemann and Raue, 2016) - that may affect the amount or type of data required.

Models don't need a ton of data to be useful—but they need the appropriate data. Sometimes what is a traditional, convenient, or intuitive design to an experimental biologist is not appropriate or sufficient for the modeling approach. For example, when addressing questions of how a range of a stimulus impacts behavior, it would be better to use a model to determine where interesting or limiting behavior might occur. It is common for modelers to be humorously critiqued for asking for impossible data—communication is required to establish the happy medium between the two perspectives and maximize our science. If a model is sufficiently precise it can describe the relationship that will appear in the data we do have, rather than the data we wish we had.

### 9. Beyond Specialization: Plant Computational Biology as a Discipline

The issue of improperly designed and implemented experiments is a well-known problem in statistics. Far from being an esoteric concern, improper experimental design limits statistical power and depth of inference. Even scientists who are careful with their analyses may run into problems. If an experiment is poorly planned or executed, computational analyses (especially toolboxes or software) will often spit out something. Although mechanistic mathematical models are rarely applied as black boxes, they can be misused in other ways. The quality of a model depends on the practical implications of those flaws for prediction, inference, or decision making. We need plant biologists to be able to evaluate the purpose, utility, and basic practices involved in modeling.

## Conclusion

Computational thinking is a fundamental skill for plant biologists (Wing, 2006; Schatz, 2012). It complements the theoretical nature of biology and how we understand how things work through the process of abstraction (Wing, 2006). With education and increased access to computational resources, mathematical and computational methods will become more common throughout plant biology. The field of plant computational biology meets this need, where applied mathematical biologists and computational biologists are experts in both mathematical and computational tools, and their applications to plant biology. We urge plant biologists interested in enhancing their research with computational modeling to meet our challenges, appreciate the science and the specialist nature of modeling, and start collaborative conversations with patience.

## Author Contributions

RD conceived of the work. RD and SO wrote the initial draft. RD, SO, AJ, M-FL, DF, AH, S-HS, AN, and AB wrote and edited the manuscript.

## Conflict of Interest

The authors declare that the research was conducted in the absence of any commercial or financial relationships that could be construed as a potential conflict of interest.

## Footnotes

^1^The term “mechanistic” is often used to describe models that incorporate a plant's physiological and regulatory mechanisms (Spitters, 1990; Jones et al., 2003; Keating et al., 2003; Estes et al., 2013). This definition of “mechanistic” is distinct from the concept of mathematical abstraction discussed in this review. These two usages of the term are not mutually exclusive, however, as some models use mechanistic mathematical models related to plant regulatory mechanisms (Roodbarkelari et al., 2010; Dreyer et al., 2019).

^2^*In Silico* Plants. Available online at: https://academic.oup.com/insilicoplants

^3^http://copasi.org/

^4^https://vcell.org/

^5^http://www.landis-ii.org/

^6^NIMBioS Investigative Workshops. Available onlie at: http://www.nimbios.org/workshops/workshop_calendar
